# Current and Novel Antiplatelet Therapies for the Treatment of Cardiovascular Diseases

**DOI:** 10.3390/ijms222313079

**Published:** 2021-12-03

**Authors:** Georges Jourdi, Marie Lordkipanidzé, Aurélien Philippe, Christilla Bachelot-Loza, Pascale Gaussem

**Affiliations:** 1 Research Center, Montreal Heart Institute, Montreal, QC H1T 1C8, Canada; marie.lordkipanidze@umontreal.ca; 2 Faculty of Pharmacy, Université de Montréal, Montreal, QC H3T 1J4, Canada; 3 INSERM, Innovations Thérapeutiques en Hémostase, Université de Paris, F-75006 Paris, France; aurelien.philippe@aphp.fr (A.P.); christilla.bachelot-loza@u-paris.fr (C.B.-L.); 4 Service d’Hématologie Biologique, AP-HP, Hôpital Européen Georges Pompidou, F-75015 Paris, France

**Keywords:** acute coronary syndrome, aspirin, atherothrombosis, bleeding, cardiovascular disease, dual antiplatelet therapy, P2Y_12_ receptor antagonists, platelets

## Abstract

Over the last decades, antiplatelet agents, mainly aspirin and P2Y_12_ receptor antagonists, have significantly reduced morbidity and mortality associated with arterial thrombosis. Their pharmacological characteristics, including pharmacokinetic/pharmacodynamics profiles, have been extensively studied, and a significant number of clinical trials assessing their efficacy and safety in various clinical settings have established antithrombotic efficacy. Notwithstanding, antiplatelet agents carry an inherent risk of bleeding. Given that bleeding is associated with adverse cardiovascular outcomes and mortality, there is an unmet clinical need to develop novel antiplatelet therapies that inhibit thrombosis while maintaining hemostasis. In this review, we present the currently available antiplatelet agents, with a particular focus on their targets, pharmacological characteristics, and patterns of use. We will further discuss the novel antiplatelet therapies in the pipeline, with the goal of improved clinical outcomes among patients with atherothrombotic diseases.

## 1. Introduction

Atherothrombotic events remain a leading cause of mortality and disability worldwide [1]. Platelet activation plays a fundamental role in mediating these ischemic diseases, making antiplatelet therapy central to their therapeutic management. During the past several years, many oral and intravenous antiplatelet agents have been developed. They have presented an escalating potency to prevent and treat atherothrombotic events encompassing cardiovascular, cerebrovascular and peripheral artery diseases. However, they have concomitantly increased clinically-relevant bleeding complications. Therefore, efforts are now focusing on identifying new platelet targets and developing novel therapeutic approaches with the aim to target receptors and pathways implicated in thrombotic process while preserving normal hemostatic platelet functions.

This manuscript provides an overview of the antiplatelet agents currently available, details their pharmacological properties and clinical indications, and discusses the current landscape of emerging antiplatelet targets and agents in development, particularly those undergoing clinical trials. 

## 2. Platelet Physiology

Platelets are the major cell components of the hemostatic system that aim to minimize blood loss by forming together with crosslinked fibrin a hemostatic plug following vascular injury. They are small anucleate cells (2–4 µm in diameter) produced by megakaryocytes mainly in the bone marrow and in the lung and are released into blood, where they circulate for 7–10 days in humans, after which they are eliminated in the spleen and liver [2]. Approximately 1 × 10^11^ platelets are released into the circulation every day, where their RNA content progressively reduces along with the loss of surface glycoproteins (GPs) sialic acid residues promoting their clearance [3].

**Figure 1:**
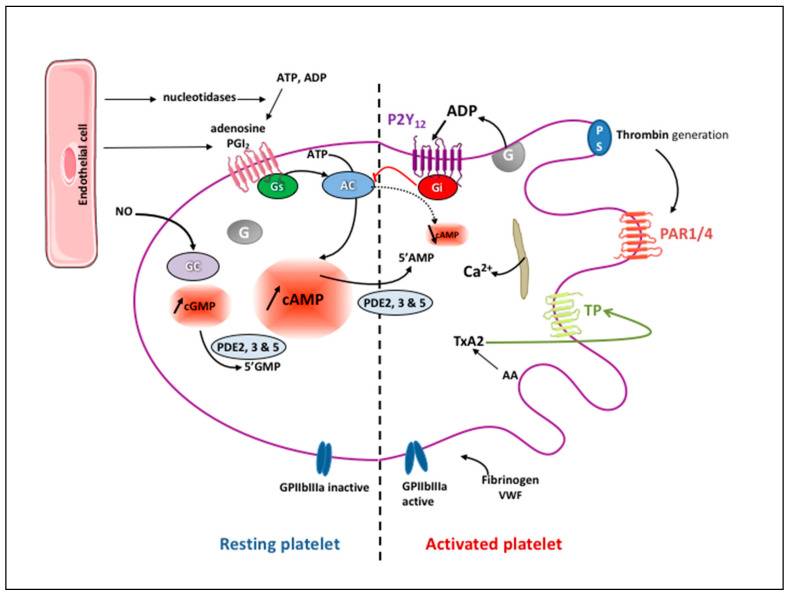
Major signaling events and responses in resting and activated platelets. Under physiological conditions, endothelial cells release nucleotidases that degrade adenosine di- and tri-phosphate (ADP and ATP, respectively) to adenosine. They also secrete prostacyclin (also known as prostaglandin I2; PGI2) and nitric oxide (NO). NO and PGI2 as well as adenosine activate within platelets guanylyl (GC) and adenylyl cyclases (AC) respectively, increasing intra-platelet cyclic guanosine and adenosine 3′,5′-monophosphate (cGMP and cAMP, respectively) which are powerful platelet inhibitors maintaining the glycoprotein (GP) IIbIIIa, also called integrin αIIbβ3, in its inactive form. cAMP and cGMP are subsequently hydrolyzed by phosphodiesterases (PDE) limiting their intracellular levels. Following platelet activation, arachidonic acid (AA) produced from membrane phospholipids upon the action of phospholipase A2 is metabolized in thromboxane A2 (TXA2) that activates the Thromboxane Prostanoid (TP) receptor. ADP, by activating P2Y12 receptor, induces an inhibition of the AC that decreases cAMP synthesis. Following coagulation activation, generated thrombin cleaves its receptors on platelet surface, i.e., the protease-activated receptors (PAR1 and PAR4), resulting in their activation. TP, P2Y12, and PAR activation increases the cytosolic Ca2+ level and induces a conformational change of αIIbβ3 on the platelet surface that links its substrates, mainly fibrinogen and the von Willebrand factor (VWF), resulting in platelet aggregation.

Physiologically, the vascular endothelium inhibits platelet activation in the circulation via (i) the release of nitric oxide (NO) and prostaglandin I_2_ (PGI_2_, prostacyclin), (ii) the expression of ectonucleotidases, which degrade adenosine tri- and di- phosphate (ATP and ADP, respectively) leading to the production of adenosine, and (iii) the expression of thrombomodulin, which binds thrombin and inhibits its prothrombotic effects [4]. PGI_2_ and NO activate adenylyl and guanylyl cyclases within platelets, respectively, thus increasing intra-platelet cyclic adenosine 3′,5′-monophosphate (cAMP) and cyclic guanosine 3′,5′-monophosphate (cGMP). Both cAMP and cGMP activate protein kinases (PKA and PKG) that phosphorylate specific substrates (i.e., phosphodiesterases (PDE) 3A and 5A, Rap1b, IP3 receptor, filamin, vasodilator-stimulated phosphoprotein, etc.), thus interfering with their own synthesis. Accumulation of cAMP and cGMP also hinders signaling induced by platelet receptor agonists, through among other factors, impaired cytosolic Ca^2+^ elevation and cytoskeletal reorganization [5]. Three PDE isoforms, namely, PDE2, PDE3, and PDE5, catalyze the hydrolysis of cAMP and cGMP to inactive 5′-AMP and 5′-GMP, thereby limiting the intracellular levels of cyclic nucleotides (Figure 1) [6]. 

Following vascular injury, platelets roll on the sub-endothelium via the interaction between the GPIb-V-IX integrin and the high-molecular-weight von Willebrand factor (VWF) of the sub-endothelium [7]. Platelets are stabilized as they adhere to VWF via a second receptor, GPIIbIIIa (also called integrin αIIbβ3), and to collagen receptors GPIaIIa (also called integrin α2β1) and GPVI. Signaling through these receptors, which involves multiple small G-protein regulators, SRC-family kinases, and serine/threonine protein kinases, leads to the activation of phosphoinositide 3-kinase (PI3K) and PLCγ followed by Ca^2+^ release into the cytoplasm [8]. Ca^2+^ and protein kinase-dependent activation of cytosolic phospholipase A2 (PLA2) within activated platelets leads to the synthesis and secretion of thromboxane A2 (TXA2) through the release of arachidonic acid (AA) from membrane glycerophospholipids and transformation into TXA2 by the sequential action of cyclooxygenase-1 (COX-1) and TXA2 synthase. TXA2, in turn, activates platelets in an autocrine and paracrine fashion via the thromboxane receptor (TP). Activation of the TP stimulates PLCβ via G_αq_ proteins, inducing Ca^2+^ release into the cytoplasm, PKC activation, and its interaction with G_α12/13_ proteins. It also triggers Rho-associated protein kinase (ROCK) activation, which is involved in platelet shape change and spreading. Besides being a potent platelet activator, TXA2 exerts a significant vasoconstrictor effect [9]. 

**Figure 2:**
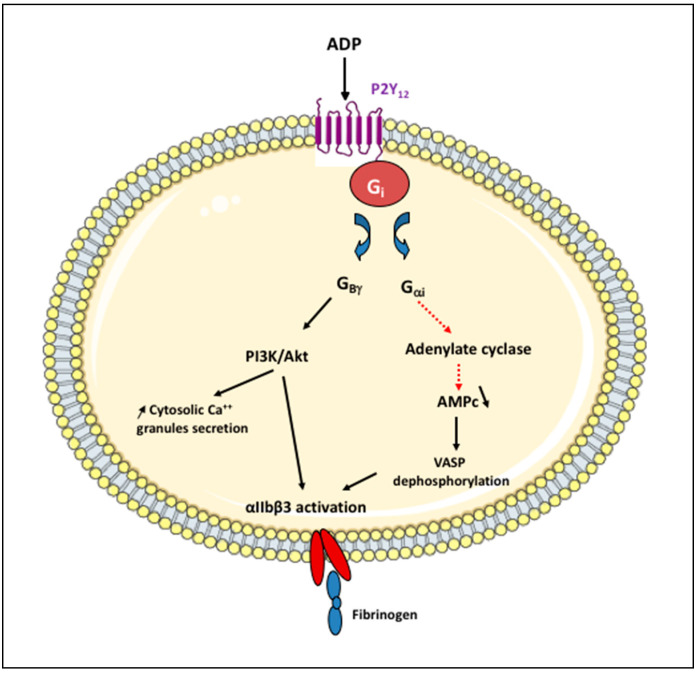
P2Y12 receptor signaling pathways in platelets. ADP acts as a soluble agonist of P2Y12 receptor. It is a Gi-protein-coupled receptor consisting of a single polypeptide chain of seven transmembrane α helices. Its activation inhibits via the Gαi subunit, the adenylate cyclase-mediated signaling, which decreases the cyclic adenosine 3′,5′-monophosphate (cAMP) levels and results in the dephosphorylation of some cytoskeletal proteins, including vasodilator-stimulated phosphoprotein (VASP). P2Y12 receptor activation also stimulates the phosphoinositide 3-kinase (PI3K) via Gβγ protein complex resulting in Akt stimulation, which activates a number of downstream substrate proteins thereby increasing the cytosolic Ca^2+^ levels and inducing granule secretion. Both pathways ultimately lead to a conformational change in the glycoprotein (GP) IIbIIIa (also known as integrin αIIbβ3) on the platelet surface, which links fibrinogen, resulting in platelet aggregation.

Human platelets contain three types of storage granules: α-granules, dense granules, and lysosomes. Dense granules contain small molecules such as ADP, ATP, serotonin, Ca^2+^, pyrophosphate, and polyphosphate as well as the lysosomal membrane proteins CD63 and lysosome-associated membrane protein (LAMP) 1 and 2 [10,11]. Following activation, platelets secrete their granular content including ADP, which acts as a soluble agonist binding to two purinergic receptors on platelets consisting of a single polypeptide chain of seven transmembrane α helices, P2Y_1_ and P2Y_12_. P2Y_1_ associates with G_αq_ to regulate platelet shape change and induce an initial weak transient phase of aggregation [12]. P2Y_12_ is a G_i_-protein-coupled receptor. Its activation inhibits G_αi,_ adenylate cyclase-mediated signaling, thus decreasing the cAMP level, and stimulates PI3K via the G_βγ_ protein complex, resulting in Akt stimulation, which activates a number of downstream substrate proteins, ultimately leading to platelet activation (Figure 2). ADP is hydrolyzed to AMP by CD39 present on the endothelial cell surface and then to adenosine by CD73. Adenosine stimulates A_2A_ and A_2B_ platelet surface receptors that activate adenylyl cyclase, increasing the intra-platelet cAMP level, which leads to platelet inhibition (Figure 1). A part of extracellular adenosine is internalized into red blood cells and platelets via a membrane-bound channel, the type 1 equilibrative nucleoside transporter (ENT1), to prevent excessive platelet inhibition [9].

Platelet activation results in a conformational change of GPIIbIIIa (or integrin αIIbβ3), from a low-affinity to a high-affinity state for fibrinogen, but also for VWF and fibronectin, facilitating platelet aggregation and activation [13]. This pathway involves CalDAG–GEFI (Ca^2+^- and diacylglycerol-regulated guanine nucleotide-exchange factor and Rap1b) and cytoskeleton-linked signaling molecules (kindlin, talin, and filamin). Ligand-bound GPIIbIIIa generates outside-in signaling events that mediate cytoskeletal reorganization and platelet spreading. It is also critical for platelet-mediated clot retraction, a process that helps seal the injury site and initiates wound healing [14]. 

Activated platelets release growth factors, chemokines, coagulation factors, RNA species, and extracellular vesicles. They also interact with leukocytes and the coagulation system, thus mediating thrombo-inflammation. Activated platelets swell and expose phosphatidylserine at their membrane surface as a result of a prolonged Ca^2+^-dependent signaling, thus leading to membrane ballooning. This event results from the activation of the Ca^2+^-activated ion channel–phospholipid scramblase, anoctamin-6 (ANO6, also known as TMEM16), and the intracellular protease calpain 2. Membrane ballooning, leading to platelet microvesicles, increases the surface area of the phosphatidylserine-exposing platelets, which likely serves to enhance their capacity for coagulation factor binding [15]. Thrombin generated through coagulation activation also potently activates platelets by cleaving the extracellular N-terminus of two G _q_ protein-coupled receptors, so-called protease-activated receptors (PAR1 and PAR4), which expose the novel C-terminal tethered ligands SFLLRN and AYPGKF, respectively. While PAR1 is activated by sub-nanomolar concentrations of thrombin, PAR4 activation requires a 10-fold higher concentration of thrombin [16]. PAR1 and PAR4 both transduce signals through G_q_ and G_12/13_. The activation of the former stimulates the formation of inositol triphosphate (IP3) and diacylglycerol, which induce intracellular Ca^2+^ mobilization and PKC activation, respectively. This pathway regulates various platelet responses including granule secretion, integrin activation, and platelet aggregation. The activation of G_12/13_ mediates Rho guanine nucleotide exchange factors and RhoA signaling pathways, which control platelet shape change [9]. Other platelet-adhesive receptors such as integrin α6β1, which interacts with laminin, and GPIV (CD36), which interacts with thrombospondin are less potent in leading to thrombus formation than the previously mentioned receptors, but still mediate platelet adhesion [3].

## 3. Currently Available Antiplatelet Agents: Targets and Pharmacological Characteristics

**Figure 3:**
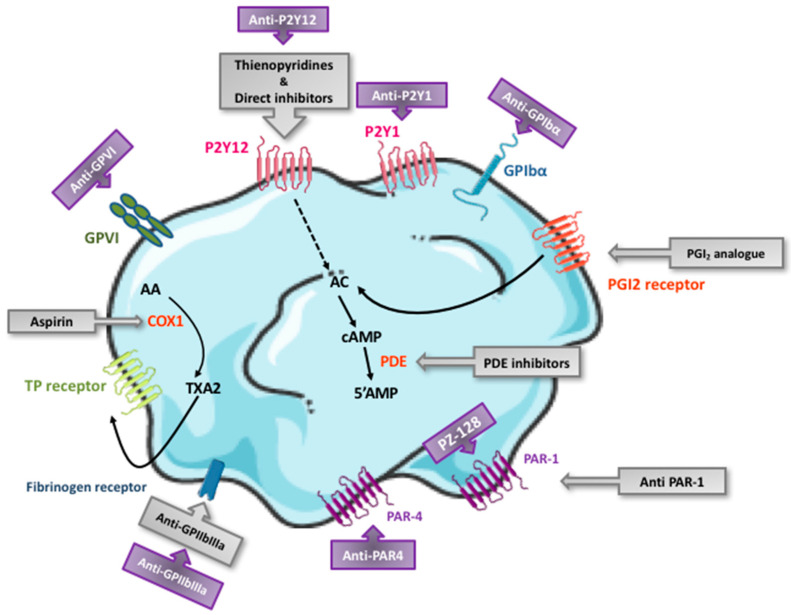
Targets of current and emerging antiplatelet therapies. The currently available antiplatelet drugs (in grey) essentially act by preventing the formation of secondary messengers (cyclooxygenase-1 [COX1] inhibitors), by interacting with intracellular signaling pathways (phosphodiesterases [PDE] inhibitors and the prostacyclin [PGI_2_] analogue), by blocking membrane receptors (P2Y_12_ receptor antagonists and the protease-activated receptor PAR1 antagonist), or by inhibiting platelet aggregation (glycoprotein [GP] IIbIIIa inhibitors). Novel antiplatelet drugs in development (in purple) are mainly directed against platelet glycoproteins such as GPVI, GPIbα, and GPIIbIIIa or block membrane receptors such as the 2 purinergic receptors P2Y_12_ and P2Y_1_ as well as the receptors PAR1 and PAR4. Other drugs directed against different platelet-activation processes, such as adhesion, signaling and pro-coagulant activity, are also under development.

The currently available antiplatelet drugs act by preventing the formation of secondary messengers (COX-1 inhibitor), by interacting with intracellular signaling pathways (PDE inhibitors and the PGI2 analogue), by blocking membrane receptors (P2Y12 receptor antagonists and the PAR1 antagonist), or by inhibiting platelet aggregation (GPIIbIIIa inhibitors) per se (Figure 3). Their main pharmacological characteristics are summarized in Table 1. 

### 3.1. Aspirin

In 1956, aspirin, used as an anti-inflammatory agent, turned out to protect from heart attacks [17]. Several years later, it was uncovered that it irreversibly inhibits COX-1 by acetylating a serine residue at position 529, which inhibits the access of AA to the active center of COX-1 and prevents the generation of prostaglandin G2 and H2 and thereby, TXA2 synthesis for the duration of the platelet lifespan [18,19,20,21]. Moreover, aspirin acetylates lysine residues on fibrinogen, thus enhancing fibrin clot permeability and clot lysis [22]. 

It is noteworthy that aspirin also inhibits COX-2 expressed in 8–10% of circulating platelets by acetylating a serine residue at position 516. However, aspirin is 170-fold more potent at inhibiting COX-1 than COX-2 [23,24]. Its full antiplatelet effect is obtained at a low dose of 75–100 mg/day [25], commonly prescribed for long-term prevention of cardiovascular disease (CVD). COX-2 inhibition is mainly observed with higher doses (>500 mg/day), resulting in an increased burden of aspirin side effects, particularly gastrointestinal bleeding without incremental antiplatelet effect [26]. 

Aspirin is most often orally administered as enteric-coated tablets. It can be intravenously injected (in Europe) or administered as chewable tablets (in North America), particularly in the first phase of acute coronary syndromes (ACS). A single aspirin dose administered in healthy volunteers showed a full inhibition of AA-induced platelet aggregation 5 min after 250 mg IV administration and 40 min after 100 mg oral administration [27]. The time to steady-state platelet inhibition after an orally administered maintenance dose is one day. A loading dose of 250–300 mg is indicated in this context followed by a daily maintenance dose of 75–100 mg as recommended by the European Society of Cardiology (ESC) or 81–325 mg as recommended by the American College of Cardiology [28,29,30,31]. Remarkably, no significant differences in cardiovascular events or major bleeding was observed in the recently published ADAPTABLE trial between patients with established CVD receiving either 81 mg or 325 mg of aspirin once daily [32]. 

Owing to its short half-life and the increased platelet turnover in some patients such as those having diabetes mellitus (DM) or essential thrombocythemia, a significant proportion of platelets may escape 24-h inhibition by once-daily aspirin, which can be re-established by twice-daily dosing [33,34]. Although the recovery of normal platelet functions is achieved 5–7 days after therapy cessation, a discontinuation of aspirin therapy only three days before high bleeding-risk procedures is recommended [35], whereas it can be continued around the time of most elective surgeries [36]. 

Non-compliance to aspirin is by far the leading cause of insufficient inhibitory effects [37]. Increased platelet turnover may be involved in the inadequate suppression of platelet aggregation while on aspirin therapy as previously mentioned [38,39,40,41,42], along with the reduced bioavailability of enteric-coated formulations in some patients [43] and the lack of specificity of most laboratory assays used to assess the biological resistance to aspirin therapy [44]. Rare genetic polymorphisms and certain drug interactions may also be associated with higher levels of serum thromboxane B2 (TXA2 stable metabolite in serum) and residual platelet aggregation [45]. This is particularly the case when aspirin is co-administered with a non-steroidal anti-inflammatory drug (NSAID), owing to the competitive inhibition of the access to the acetylation site of platelet COX-1 [45]. Therefore, these drugs should be administered several hours after aspirin intake. 

Moreover, aspirin is a substrate for the organic anion unidirectional transporter called the multidrug resistance protein-4 (MRP4); thus, it can be extruded from platelets [46,47]. While the aspirin effect on COX-1 is little related to MRP4-mediated transport in healthy individuals, a higher expression of platelet MRP4 may be observed in patients with CVD, probably owing to the genomic modulation of megakaryocytes induced by the aspirin treatment itself [48]. MRP4 pharmacological inhibition, via dipyridamole for instance, was shown to efficiently enhance aspirin action in these patients [47]. 

### 3.2. P2Y12 Receptor Antagonists

The P2Y_12_ receptor antagonists include two drug classes: the thienopyridines and the nucleoside–nucleotide derivatives. 

#### 3.2.1. Thienopyridines 

Ticlopidine was the first drug of this class commercialized in 1991, but it is no longer used in clinical practice due to numerous side effects, mainly cytopenia, gastrointestinal disorders, and allergic reactions [49]. 

**Figure 4:**
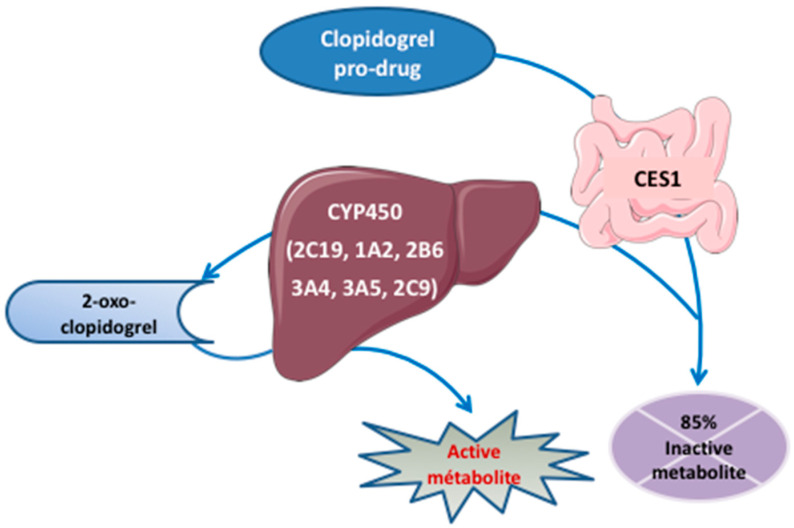
Clopidogrel metabolism pathways. Clopidogrel is extensively metabolized (85%) to a pharmacologically inactive metabolite via intestinal carboxylesterase 1 (CES1) and is eliminated in the urine and feces. The remaining 15% is metabolized by mixed-function oxidase enzymes from the CYP superfamily, first to 2-oxo-clopidogrel via CYP2C19, 1A2, and 2B6, and then to the active metabolite via CYP2C19 and, to a lesser extent, 2C9, 3A4, 3A5, and 2B6.

A second-generation thienopyridine, the pro-drug clopidogrel, entered clinical trials in 1987 and was approved in 1997. Its molecular target remained unknown until the P2Y_12_ receptor was identified as its target in 2001 [50]. Since then, it has been increasingly prescribed as an oral, irreversible, competitive P2Y_12_ receptor antagonist. Clopidogrel is administered orally and absorbed from the intestinal lumen via the ATP-dependent intestinal efflux transporter P-glycoprotein (P-gp) encoded by the ABCB1 (also called MDR1) gene. It is prescribed with a loading dose of 300–600 mg and a daily maintenance dose of 75 mg [28,29,30,31]. Whereas the maximal antiplatelet effect is obtained within 6–8 h with a 300 mg loading dose, it is attained within 2–4 h with a 600 mg dose. Its bioavailability exceeds 50%. It is subsequently extensively metabolized (85%) to pharmacologically inactive SR26334 via intestinal carboxylesterase 1 (CES1) [51] and is eliminated in the urine and feces. The remaining 15% is metabolized by mixed-function oxidase enzymes from the CYP superfamily first to 2-oxo-clopidogrel (via CYP2C19, 1A2, and 2B6) and then to the active metabolite (R130964; by CYP2C19, and, to a lesser extent, by 2C9, 3A4, 3A5, and 2B6 as well as paraoxonase 1 [PON1]). The active metabolite covalently binds the P2Y_12_ receptor and irreversibly and rapidly inhibits ADP-dependent platelet activation and aggregation (Figure 4) [52]. 

2-oxo-clopidogrel may also be partially hydrolyzed by CES1 to an inactive acid metabolite. Among the aforementioned enzymes, CYP2C19 is the most involved in clopidogrel bioconversion either in the first step (45%) or the second step (21%). The elimination half-life of the active metabolite of clopidogrel (i.e., R130964) is estimated to be approximately 30 min. The time to onset of action after a loading dose is approx. 2–6 h, whereas the time to steady-state platelet inhibition after maintenance dosing is approx. five days. The time to platelet function recovery is approx. seven days while it is proposed to be discontinued five days before surgery [35] except in the case of intracranial surgery, where two additional days free of clopidogrel therapy should be considered. 

Up to 10% of patients experience recurrent ischemic events at 12 months after ACS despite receiving dual antiplatelet therapy (DAPT) combining aspirin and clopidogrel [53]. In addition, 4–34% of patients receiving clopidogrel continue to present a high on-treatment platelet reactivity [54,55]. These challenges led to the development of a third-generation thienopyridine, prasugrel, marketed as a pro-drug in 2009. Its active metabolite irreversibly and competitively inhibits ADP-induced platelet aggregation faster, more consistently, and to a higher degree than clopidogrel [56,57]. Prasugrel is prescribed with a loading dose of 60 mg (reduced to 20 mg in Japan), and its daily maintenance dose is 10 mg, which is reduced to 5 mg or even 3.75 mg in Japan, and in patients aged older than 75 years or with a body weight less than 60 kg in certain countries [28,29,30,31,58]. It is absorbed from the intestinal lumen. Unlike clopidogrel, its absorbance does not extensively depend on P-gp activity [59]. Its bioavailability exceeds 75%. Once absorbed, inactive prasugrel is hydrolyzed to the thiolactone metabolite R-95913 by the esterase CES2 predominantly expressed in the intestine. R-95913 is then metabolized to the active metabolite R-138727 with a one-step activation involving the CYP450 superfamily, mainly CYP2B6 and 3A4 [60,61]. The latter is a predominant CYP isoform in the intestine, which may explain, at least partially, the rapid bioavailability of prasugrel active metabolite [62]. As prasugrel relies less on CYP-mediated metabolism, it produces less response variability than clopidogrel. Its elimination half-life is approximately 4 h and that of its active metabolite (i.e., R-138727) is 30–60 min. The time to onset of action is estimated around 30 min following a loading dose. The time to steady-state platelet inhibition after maintenance dosing is approx. three days, and the time to platelet function recovery is seven to ten days [63]. If discontinuation of antiplatelet therapy is indicated before the procedure, a last intake of prasugrel seven days before surgery is proposed [35] except in case of intracranial surgery where 10 days of prasugrel therapy cessation should be considered.

#### 3.2.2. ATP Analogues

Ticagrelor and cangrelor belong to a new generation of reversible P2Y_12_ receptor antagonists. Ticagrelor is an oral ATP analogue (cyclopentyl-triazolo-pyrimidine) that binds the P2Y_12_ receptor at a distinct site from that of ADP. It was approved in Europe and USA in 2010 and 2011, respectively. Unlike clopidogrel and prasugrel, ticagrelor does not require metabolic activation and achieves a faster, more potent and more predictable antiplatelet effect than clopidogrel [64]. Additionally, ticagrelor inhibits adenosine reuptake via the ENT1 transporter in erythrocytes and platelets, which may improve its antiplatelet effect [65]. Ticagrelor is rapidly absorbed from the intestinal lumen with a possible involvement of P-gp and thereafter exerts a direct antiplatelet effect [66]. Its bioavailability (36%) is less important than that of clopidogrel and prasugrel. It is metabolized mainly by CYP3A4 and to a lesser extent by CYP3A5 to an active metabolite, AR-C124910XX, which appears in circulation within 3 h following drug intake and represents 30–40% of circulating active drug. Ticagrelor is also a mild inhibitor of CYP3A4. It is thus contraindicated in association with strong CYP3A4 inhibitors (such as ketoconazole and ritonavir). Both ticagrelor and AR-C124910XX are equipotent and act at a distinct site on the P2Y_12_ receptor to prevent ADP-mediated platelet activation [67]. The onset of ticagrelor antiplatelet action after a loading dose is 30 min. Ticagrelor and AR-C124910XX have an elimination half-life of 7–9 h and 9–12 h, respectively. The time to steady-state platelet inhibition after maintenance dose is less than five days, and the time to platelet function recovery is approximately 3–5 days. A last intake of ticagrelor five days before surgery is commonly proposed [35], with two additional days free of antiplatelet therapy in case of intracranial procedures. The ESC guidelines proposed the reduction of ticagrelor-free period to three days especially before cardiac surgery [31]. 

Cangrelor has been commercialized since 2015 as an intravenous ATP analogue that reversibly inhibits P2Y_12_ receptor. Administered intravenously, cangrelor has an ultra-short half-life of 3–6 min and rapid onset and offset effects allowing a rapid recovery of platelet function within 1 h [68]. The recommended dosage of cangrelor in ACS patients is 30 µg/kg IV bolus (administered in less than 1 min), followed immediately by a 4 µg/kg/min IV infusion for at least 2 h or the duration of percutaneous coronary intervention (PCI) [69,70]. It is an interesting therapeutic option in ACS patients not pre-treated with an oral P2Y_12_ receptor antagonist who need urgent PCI [71] or those who require DAPT bridging before surgery [72]. It may also be the optimal antiplatelet agent in patients who require immediate platelet inhibition and who are unable to swallow [73]. 

### 3.3. GPIIbIIIa Inhibitors

GPIIbIIIa inhibitors are commercialized as IV antiplatelet agents that block the association of fibrinogen and VWF to the GPs on the platelet surface. They have been introduced to enable fast platelet aggregation inhibition and reduce the risk of ischemic complications (although they do not inhibit platelet activation) associated with ACS. Abciximab was the first agent of this class, consisting of a chimeric (mouse and human) monoclonal antibody, approved in 1994 [74] then withdrawn from the market in 2019 following the interruption of its production by Janssen laboratories. 

Tirofiban is a non-peptide derivative of tyrosine mimicking the fibrinogen-binding sequence within GPIIbIIIa, and eptifibatide is a cyclic heptapeptide with a lysine–glycine–aspartic acid (RGD) motif derived from a protein found in the venom of rattlesnakes that also mimics the fibrinogen-binding sequence. Both are small molecules that inhibit GPIIbIIIa in a competitive manner with a stoichiometric ratio > 100:1, thereby acting as potent antiplatelet drugs [74]. 

Eptifibatide is administered as a bolus of 180 µg/kg (with another 180 µg/kg later if PCI is performed) and an infusion of 2 µg/kg/min (for 18 h), and tirofiban is administered as a bolus of 25 µg/kg (over 3 min.) followed by an infusion of 0.15 µg/kg/min (up to 18 h) [69]. Their half-lives are 2.5 and 2 h and the time to steady-state platelet inhibition is ≤15 min and 20–40 min, respectively. The time to platelet function recovery is 4–8 h after stopping eptifibatide and tirofiban infusion [75,76]. 

### 3.4. Phosphodiesterase Inhibitors

Dipyridamole (2,6-bis (diethanolamino)-4,8-dipiperidino- pyrimido 5,4-d pyrimidine) was synthesized about half a century ago and initially used as a coronary vasodilator. Its antiplatelet activity was subsequently discovered in an in vivo experiment in rabbits [77]. It inhibits platelet PDE3 and especially PDE5. In endothelial cells, it induces the synthesis and release of PGI_2_. Dipyridamole also increases the extracellular levels of adenosine through the inhibition of its reuptake by red blood cells. Moreover, its potential to scavenge peroxy radicals, to reduce innate inflammation, and to increase interstitial adenosine levels seems to be more important than its adenosine- and PGI_2_-mediated antithrombotic effect for the prevention of vascular and tissue damage [78]. Furthermore, dipyridamole inhibits MRP4 mediated transport of aspirin, thus increasing its entrapment within platelets, which partially explains the therapeutic synergism of dipyridamole with aspirin [47]. All of the above findings support the use of dipyridamole in some patients, usually in association with aspirin. Dipyridamole is administered orally at the dose of 75–100 mg four times per day. Its bioavailability is around 70% and it is largely bound to proteins (99%) in circulation. Dipyridamole is metabolized in the liver where it is conjugated as a glucuronide and excreted in the bile. Its elimination half-life is approx. 10–12 h. The time to steady-state platelet inhibition is approx. 4–7 days [79], whereas the time to onset of action and the time to platelet function recovery following its cessation remain to be determined. 

In 1988, cilostazol, 6-[4-(1-cyclohexyltetrazol-5-yl)-butoxy]-3,4-dihydro-1H-quinolin-2-one, was first marketed in Japan as a selective inhibitor of PDE3A (the main subtype of PDE3 expressed in platelets) that also inhibits adenosine cellular uptake. Moreover, cilostazol exerts a vasodilatory effect by inducing vascular smooth muscle cell relaxation via PKA-mediated inhibition of myosin light-chain kinase and activation of calcium-activated potassium channels [80] and improves endothelial function by decreasing endothelial oxidative stress via the suppression of nicotinamide adenine dinucleotide phosphate (NADPH) oxidase (NOX)-2 expression [81]. The recommended dosage of cilostazol is 100 mg twice daily. It is rapidly absorbed and reaches peak plasma concentrations at approximately 2.4 h after oral administration. It is largely bound to proteins (95–98%), primarily albumin, in circulation. Cilostazol is predominantly metabolized in the liver by CYP3A4 and, to a lesser extent, by CYP2D6 and CYP2C19. The metabolites and <1% of the administered dose in its unchanged form are largely excreted in urine [82]. The main active metabolite of cilostazol is 3,4-dehydro-cilostazol. Its antiplatelet effect is 15-fold more potent than that of cilostazol [82]. Cilostazol elimination half-life is estimated to be approximately 11–13 h [82], whereas the time to onset of action following its administration remains to be determined. A potential benefit of cilostazol over conventional antiplatelet therapy is the relatively short time of platelet function recovery estimated at around 12–16 h following its discontinuation [83]. 

### 3.5. Prostacyclin Analogue

Iloprost is a stable analogue of epoprostenol (a potent prostanoid also called prostacyclin or PGI_2_); it increases platelet cAMP levels, thus acting as an intravenous reversible antiplatelet agent. Its potent but short-lived effects make it well-suited for certain therapeutic niches such as the management of intraoperative platelet activation [84]. Moreover, iloprost is an arterial vasodilator (through cAMP level enhancement in vascular smooth muscle cells), which increases its therapeutic value for systemic administration but makes hypotension a limitation of this therapy [84]. Most patients tolerate infusion rates of up to 2 ng/kg/min. It is completely metabolized by β-oxidation; the metabolites are predominantly (70%) eliminated by renal excretion, and another 12–17% are eliminated by fecal excretion. Elimination of iloprost is biphasic, with an initial half-life of distribution of 4 min and elimination half-life of approximately 30 min. Patients with severe hepatic disease or renal disease requiring maintenance hemodialysis have a 2–3-fold reduction in iloprost clearance and a substantial elevation of plasma drug concentrations [84]. The time to steady-state platelet inhibition after maintenance therapy as well as that to platelet function recovery remains to be established. 

### 3.6. PAR1 Antagonist

Vorapaxar is an oral PAR1 reversible antagonist derived from a natural product, himbacine [85]. It is the last class of antiplatelet agents that was approved by the Food and Drug Administration (FDA) in 2014 for the reduction of thrombotic cardiovascular events in patients with a history of heart attack or with peripheral artery disease (PAD). Vorapaxar has not yet gained the European Medicines Agency approval [86]. Although very rarely used in clinical practice, vorapaxar is administered as a loading dose of 40 mg, followed by a daily maintenance dose of 2.5 mg, in addition to DAPT combining aspirin and clopidogrel. It has a high bioavailability (approximately 98%) and a very long elimination half-life (5–13 days) which results in a long time to normal platelet function recovery after therapy cessation, estimated at approximately 4–8 weeks [87]. 

## 4. Indications of the Currently Available Antiplatelet Agents

Antiplatelet agents are mainly indicated for the treatment and prevention of atherothrombotic diseases including ACS, stable coronary artery disease (CAD), PAD, ischemic stroke, and transient ischemic attack (TIA). Somewhat less frequently, they can also be used in other pathologies such as pre-eclampsia and myeloproliferative syndromes. Globally, aspirin (the COX-1 inhibitor) and P2Y_12_ receptor antagonists are by far the most commonly prescribed. Needless to say, treatment strategies may vary across countries, particularly with regard to the choice of molecules, dosage, and treatment duration. 

### 4.1. Primary Prevention of CVD

Aspirin efficacy and safety in the primary prevention of CVD remain controversial [88,89,90]: while it may be considered in USA in a subset of patients aged 40 to 70 years at high ischemic and no increased bleeding risks [91,92], it may only be proposed in DM patients with high ischemic risk in Europe [93]. No trial suggesting a definite role of the other antiplatelet agents in the primary prevention of CVD has been published to date. 

### 4.2. Acute Coronary Syndrome

In the setting of ACS, aspirin is indicated in association with a P2Y12 receptor antagonist for secondary prevention of major adverse cardiovascular events for 6–12 months depending on patient’s bleeding risk [31,94,95]. This duration can be extended beyond 12 months (up to three years) in patients at high risk of ischemic events who have tolerated DAPT well. In medically-managed ACS patients, ticagrelor is indicated as a P2Y_12_ receptor antagonist in association to aspirin, whereas in ACS patients undergoing PCI without any history of stroke (either ischemic or hemorrhagic), prasugrel or ticagrelor may be prescribed with no preference for one over the other. Prasugrel may be preferred over ticagrelor post PCI in patients with non-ST-elevation (NSTE) ACS [96], and clopidogrel may be a favorable alternative to ticagrelor or prasugrel in patients aged 70 years or older presenting with NSTE-ACS, as fewer bleeding events and no increase in the combined endpoint of all-cause death, myocardial infarction, stroke, and bleeding were recorded in the POPular AGE trial [97]. It is worth mentioning that clopidogrel is currently the commonly used P2Y_12_ receptor antagonist in ACS patients who have undergone thrombolysis. GPIIbIIIa inhibitors are very rarely prescribed in an ACS context owing to concerns regarding bleeding and the introduction of potent oral P2Y_12_ receptor antagonists. They can still be considered as a bailout therapy in the event of angiographic evidence of a large thrombus, slow or no reflow, and other thrombotic complications in patients with ST-segment elevation myocardial infarction (STEMI) undergoing PCI or in patients with NSTE-ACS undergoing high-risk PCI without pre-treatment with oral P2Y_12_ receptor antagonists [98]. 

Following DAPT, aspirin is recommended as a single antiplatelet therapy indefinitely as it is affordable and widely available even in low-income countries. 

Special attention should be given to some patients’ population having a high ischemic risk. Clopidogrel is the only P2Y_12_ receptor antagonist that can be prescribed as part of the triple antithrombotic therapy (in association to aspirin and oral anticoagulant) [31,99,100] in patients with atrial fibrillation (AF) suffering from ACS. Triple therapy should be as short as possible: during index hospitalization or up to one or six months (depending on the patient’s ischemic and bleeding risk). This is followed by dual antithrombotic therapy (single antiplatelet agent plus oral anticoagulant) for one year after coronary stenting and then by oral anticoagulation indefinitely. As part of dual antithrombotic therapy, prasugrel is allowed in the Japanese guidelines [101] whereas ticagrelor may be an alternative to clopidogrel in patients with high ischemic and low bleeding risk according to the American and European guidelines [102,103]. In patients with mechanical heart valves undergoing PCI, a daily dose of clopidogrel in addition to vitamin K antagonist is indicated following a 1-month triple therapy that could be prolonged up to six months in patients with high ischemic risk [104]. Beyond one year of dual therapy, oral anticoagulation is currently recommended with subsequent withdrawal of antiplatelet agents. Another particular population is that of patient suffering from DM due to increased platelet reactivity seen at baseline and on-treatment in diabetic patients. Prasugrel and ticagrelor are thus preferred in these patients in association with aspirin [105,106]. 

### 4.3. Stable Coronary Artery Disease

Aspirin is commonly prescribed for secondary prevention of atherothrombotic complications in CAD patients. In patients undergoing elective stent implantation with low-to-moderate bleeding risk, it can be combined with a P2Y_12_ receptor antagonist, mainly clopidogrel, for up to six months [31,95,98]. This duration should be shortened to three months in the case of a high bleeding risk [31,95,98].

### 4.4. Peripheral Artery Disease

In patients with chronic symptomatic PAD, aspirin is commonly prescribed as a long-term single antiplatelet therapy although clopidogrel may also be prescribed [107,108]. In USA, vorapaxar may be used in addition to standard antiplatelet therapy for secondary prevention in patients with a history of myocardial infarction or symptomatic PAD without any history of stroke, TIA, or intracranial hemorrhage [109,110,111]. Cilostazol is also approved for the treatment of patients with intermittent claudication in the absence of tissue necrosis or rest pain [80,112]. Moreover, iloprost may be prescribed in severe PAD patients and in those undergoing extracorporeal circulation or for the intraoperative management of heparin-induced platelet activation.

### 4.5. Stroke and Transient Ischemic Attack

DAPT-associating aspirin with clopidogrel for up to 90 days may be prescribed in patients with recent (within 30 days) stroke or TIA attributable to severe stenosis (70–99%) of a major intracranial artery [113]. Beyond DAPT, aspirin can be prescribed for the secondary long-term prevention of stroke and TIA as a single therapy or in combination with dipyridamole [114,115]. Clopidogrel is a good alternative, particularly in patients with frequent headaches, secondary to aspirin/dipyridamole combination [115,116]. Cilostazol may also be used for secondary stroke prevention, particularly in Asian patients [117]. Randomized clinical trials are still needed to determine its usefulness in non-Asian populations. In case of moyamoya disease, extracranial large artery atherosclerosis, and aortic arch atherosclerosis, a once-daily dose of aspirin is recommended to reduce the risk of recurrent stroke [113]. 

## 5. Antiplatelet Agents under Preclinical/Clinical Development

Recurrent thrombotic events occur in one in 10 patients in the first year following ACS despite treatment with the most potent antiplatelet therapy. Currently available antiplatelet drugs have some practical challenges in a real-world setting, especially the significantly increased bleeding risk. These limitations have stimulated research interest to identify new antiplatelet targets. Following the latest advances in the understanding of thrombus formation, it is now known that the thrombotic response that regulates the growth of a propagating outer layer of the thrombus primarily involves platelets in lower activation states, the recruitment of which is less sensitive to standard antiplatelet therapy [118]. However, platelets located close to the site of arterial injury are fully activated by soluble agonists such as TXA2, ADP, and thrombin and are thus more sensitive to currently available antiplatelet agents. The challenge is that although new antiplatelet agents are expected to cause less bleeding, they should not exhibit reduced antithrombotic potency. Unlike most of the currently available antiplatelet drugs that suppress autocrine events involved in platelet aggregation, novel drugs in development are frequently directed against other platelet activation processes, such as adhesion, signaling, and pro-coagulant activity (Figure 3). Here, we highlight new antiplatelet agents that are in advanced preclinical development or have already entered into the clinical development phase (summarized in Table 2). How these potential new therapeutics will fit within the current paradigm of antiplatelet therapy and whether they will lead to safer combinations in the clinical practice remain to be determined.

### 5.1. Novel PAR1 Antagonists

PZ-128 is a membrane-tethered, cell-penetrating lipopeptide called pepducin that targets the cytoplasmic domain of PAR1 [129]. Its safety and efficacy as an antiplatelet agent were evaluated in a phase I study [119]. Intravenous administration of PZ-128 inhibited PAR1-dependent platelet activation ex vivo and did not cause bleeding. 

Apart from PZ-128, and unlike orthosteric inhibitors such as vorapaxar, which inhibit all downstream signaling of PAR1 receptor, parmodulins are non-peptidic small molecules that selectively interfere with Gq signaling downstream of PAR1. Consequently, they inhibit PAR1-mediated platelet activation and aggregation [130] without affecting the cytoprotective signaling pathways in endothelial cells [130]. No clinical trials have yet been conducted with these new agents.

### 5.2. PAR4 Antagonists

Specific antagonists of PAR4 block platelet activation induced by high thrombin concentrations while preserving PAR1 signaling. Multiple compounds including inhibitory antibodies, pepducins, and small molecules have been developed. Among the latter, BMS-986120 did not cause spontaneous bleeding in healthy volunteers but reduced thrombus growth ex vivo to a similar extent as the combination of aspirin plus clopidogrel [120]. 

A more potent molecule, BMS-986141, has entered a phase II trial among patients with history of stroke or TIA already on aspirin. However, it was terminated early for undisclosed reasons and the results are still pending. No clinical trials have yet been conducted with pepducin targeting PAR4 or PAR4 inhibitory monoclonal antibodies [131,132].

### 5.3. GPVI Antagonists

Patients with GPVI deficiency usually display only a mild bleeding phenotype, which in addition to the fact that ruptured atherosclerotic plaques rich in collagen provide a robust surface for GPVI-mediated platelet adhesion, has led to interest in targeting GPVI to reduce atherothrombosis. It is particularly promising as GPVI is expressed exclusively on platelets, thus limiting potential off-target effects. Two agents have been developed: revacept, which targets GPVI ligand collagen, and glenzocimab (ACT017), which targets the GP itself. Revacept is a fusion recombinant protein that contains the extracellular domain of GPVI fused to the Fc fragment of human IgG1 [133]. The mechanism of revacept is to act as a vascular coating that competes with platelet GPVI for binding to collagen. It dose-dependently inhibited collagen-induced platelet activation without impairing hemostasis in a phase I trial [121]. An exploratory phase II trial evaluating revacept in patients with symptomatic carotid artery stenosis was recently published. It provided valuable insights into the safety, tolerability, and efficacy of revacept in this patients’ population [134]. A second ongoing phase II trial is comparing the effect of revacept on top of DAPT in patients with stable CAD undergoing PCI [135]. Apart from revacept, ACT017, a humanized Fab fragment, also targets GPVI with high affinity and specificity [136]. The safety and tolerability profiles were reported in a phase I clinical trial [122], and a phase II trial in stroke patients is currently being carried out. 

### 5.4. C-Type Lectin-Like Receptor Inhibitors 

C-type Lectin-like Receptor (CLEC-2) is a transmembrane protein expressed mainly as a dimer on platelets and at low levels on Kupffer cells, subpopulations of circulating myeloid cells, and dendritic cells [137,138]. It is physiologically activated by podoplanin and by the oxidized product of heme and hemin released during red blood cell lysis [139,140]. It was shown to have a minor role in hemostasis in adult mice in contrast to its critical role in early cerebrovascular and respiratory system development [141,142,143,144,145]. Preclinical studies have proven its implication in inflammatory-induced thrombosis secondary to the upregulation of podoplanin in vessel walls [146]. While cobalt hematoporphyrin was shown to inhibit CLEC-2-podoplanin interaction and block tumor metastasis and thrombosis in mice, it is unlikely to undergo clinical development due to its toxicity and the lack of oral bioavailability [147]. AYP1, a high affinity mAb to human CLEC-2, was generated and used to measure CLEC-2 expression on resting and stimulated platelets. It also blocked platelet activation by podoplanin and the snake venom rhodocytin [148]. However, targeting CLEC-2 might induce thrombocytopenia [149], which should be closely monitored in future in vivo assays. That said, there are currently no clinical trials targeting CLEC-2.

### 5.5. Bruton Tyrosine Kinase Inhibitors 

Bruton tyrosine kinase (BTK) is a critical downstream signaling component of GPVI. Ibrutinib is an oral irreversible inhibitor of BTK prescribed for patients with B cell malignancies [150]. Approximately 35% of treated patients suffered from low-grade bleeding complications [151]. It was shown to inhibit in vitro platelet adhesion and aggregation under static and flow conditions [152] and to suppress GPVI-dependent thrombus formation on human atherosclerosis plaque tissue ex vivo [152]. It is currently under investigation as a novel antiplatelet agent; however, adverse effects on other cells and organs are inevitable. 

### 5.6. Inhibitors of the von Willebrand-GPIbα Axis

Five molecules are being investigated as VWF inhibitors targeting platelet functions: a DNA-based aptamer (ARC1779), two monoclonal antibodies (AJW200 and 82D6A3), and two bivalent nanobodies (caplacizumab and ALX-0681). Caplacizumab was approved by the FDA in 2019 for adult patients with acquired thrombotic thrombocytopenia purpura in combination with plasma exchange and immunosuppressive therapy [153]. It was tested in combination with DAPT in patients with acute myocardial infarction undergoing PCI. However, an excess bleeding risk was reported, which was similar to that observed with the association of DAPT and abciximab [154]. ARC1779 binds the A1 domain of VWF and dose-dependently inhibits platelet function ex vivo [123]. It reduced cerebral embolization after carotid endarterectomy in a phase II clinical trial [124]. 

Moreover, four molecules are being investigated as direct GPIbα inhibitors: two antibodies (SZ2 and h6B4-Fab), a snake toxin (anfibatide), and a fusion protein containing the amino terminus of GPIbα fused to human IgG1 Fc. A phase II clinical trial assessing the safety and efficacy of anfibatide in patients with STEMI-ACS before PCI was supposed to be completed by 2016 (NCT02495012), but no data are yet available. No other clinical trials assessing the antiplatelet effectiveness of these agents in CVD patients have started.

### 5.7. Phosphoinositide 3-Kinase-β Inhibitors

PI3Kβ plays an important role in signaling downstream of various platelet receptors such as GPIb, P2Y_12,_ and GPIIbIIIa, and is required for stable platelet adhesion under shear stress. However, PI3Kβ is also involved in other cell signaling processes, which may confer some side effects to antiplatelet molecules targeting it. Three isoform-selective PI3Kβ inhibitors, AZD6482, TGX221, and MIPS-9922, are in development. Whereas the latter two are in preclinical development, AZD6482 in combination with aspirin exhibited greater antiplatelet activity with less bleeding potential than clopidogrel plus aspirin in healthy subjects [125]. Further research is being undertaken to improve its pharmacokinetic profile and selectivity towards PI3Kβ. 

### 5.8. Protein Disulfide Isomerase Inhibitors

Protein disulfide isomerase (PDI) within platelets plays an important role in mediating platelet accumulation following vascular injury by interfering with protein folding (such as GPIIbIIIa). However, it does not interfere with the initial phases of platelet adhesion and is therefore not required for normal hemostasis [155]. Quercetin was proven to inhibit platelet cell signaling in a pilot human dietary intervention [156] and to mediate antiplatelet activity via PI3K/Akt inactivation, cAMP elevation, and vasodilator-stimulated phosphoprotein (VASP) stimulation [157]. A methylated form of quercetin, pentamethylquercetin (having a better bioavailability and metabolic stability than quercetin), decreased in vitro platelet aggregation and granule secretion induced by low-dose agonists (including ADP, collagen, and thrombin) and significantly inhibited thrombus formation in mouse models [158]. Isoquercetin, a naturally occurring 3-O-glucoside of quercetin commonly consumed in fruits, vegetables, and food supplements, showed antithrombotic potential in mice [159] and decreased D-dimer platelet-dependent thrombin generation and soluble P-selectin levels in patients with advanced pancreatic, non-small-cell lung, or colorectal cancer in phase II/III clinical study [128]. Further clinical studies are required to prove its effectiveness. Another more selective and more potent inhibitor of the PDI, ML359, has also been identified and is supposed to undergo further testing [160].

### 5.9. Novel GPIIbIIIa Inhibitors

The major disadvantage of the currently available GPIIbIIIa inhibitors is the increased risk of bleeding. Moreover, ligand-mimetic GPIIbIIIa inhibitors may induce conformational changes after binding to their target, thus potentially causing severe thrombocytopenia and paradoxical platelet activation [161]. Unlike with the long-term use of GPIIbIIIa inhibitors, the aforementioned limitation may be superseded by the short-term blockade of this GP. RUC-4 binds to the metal ion-binding site on GPIIIa, thereby inhibiting ligand binding without inducing a conformational change and thus paradoxical platelet activation [162]. In preclinical studies, RUC-4 showed high antithrombotic efficacy [162]. It can be administered by intramuscular injection, which raises the prospect of administration in pre-hospital settings. However, it inhibits both non-activated and activated GPIIbIIIa and therefore, all circulating platelets. The bleeding risk profile of this agent is yet to be established. Another promising strategy for targeting GPIIbIIIa is to inhibit only the activated isoform of this glycoprotein. Single-chain variable fragments (scFvs) directed against the active conformation of GPIIbIIIa were coupled with the ADP-hydrolyzing enzyme CD39 [163], the potent factor Xa inhibitor tick anticoagulant peptide [164], or the fibrinolytic agent urokinase [165]. All these compounds have displayed potent antithrombotic effects in preclinical models without affecting hemostasis. Clinical studies are eagerly awaited. Another interesting approach could be to specifically inhibit the early phases of this integrin outside-in signaling, such as the interaction between the intracellular domain of the β_3_ subunit and Gα_13_ with a myristoylated peptide ExE peptide motif (mP6) [166]. Further studies are required to confirm the efficacy and safety of such a novel antiplatelet approach.

### 5.10. Novel P2Y_12_ and P2Y_1_ Receptors Antagonists

Selatogrel, a novel P2Y_12_ receptor antagonist, was recently evaluated in patients with chronic coronary syndrome. It was rapidly absorbed following subcutaneous administration and attained a peak plasma concentration 30 min after a single injection, thus providing prompt, potent, and consistent platelet P2Y_12_ inhibition sustained for ≥8 h and reversible within 24 h. It was assessed in a small-sized trial (*n* = 47) that included acute NSTEMI and STEMI patients. In total, 90% of the patients with acute myocardial infarction had a profound P2Y_12_-mediated platelet inhibition (measured by VerifyNow^®^) 30 min after the injection of selatogrel 8 or 16 mg [167]. This subcutaneously administered drug may open up a promising new avenue of self-administration of an anti-P2Y_12_ receptor antagonist in patients early after the onset of myocardial infarction symptoms, which aims to reduce the ischemic time and thus to limit the size of irreversible myocardial damage. Further studies are warranted to evaluate the clinical efficacy and safety of such a novel approach in acute settings where rapid platelet inhibition is desirable, as for example in ACS patients [168,169]. Two other novel highly potent inhibitors of P2Y_12_ are under development, AZD1283 and SAR216471 [170,171], the latter being in the most advanced stage of development. It was associated with less bleeding, higher selectivity, and equivalent antithrombotic efficacy compared to ticagrelor in a rat model and is currently undergoing a phase II study (NCT03384966). 

The potential of P2Y_1_ inhibition as an antiplatelet strategy that does not significantly increase the bleeding risk has also been explored. The compound BMS-884775 demonstrated similar efficacy with less bleeding compared to prasugrel in a rabbit model [172]. Another P2Y_1_ receptor antagonist, MRS2500, was shown to prevent carotid artery thrombosis in cynomolgus monkeys [173]. Moreover, combining P2Y_1_ and P2Y_12_ receptors inhibition is also of interest and has led to the development of diadenosine tetraphosphate and other derivative compounds [174]. Of these, GLS-409 improved coronary blood flow recovery in a canine model of unstable angina, with minimal increase in bleeding time [175]. No clinical trials assessing the efficacy and safety of these novel P2Y_1_ receptor antagonists have yet been started.

### 5.11. 12-Lipoxygenase Inhibitor

Platelet 12-lipoxygenase is an oxygenase predominantly expressed in human platelets. It metabolizes AA to form bioactive metabolites (12-(S)-hydroperoxyeicosatetraenoic acid and 12-(S)-hydroxyeicosatetraenoic acid [12-HETE]) that activate platelets and induce granule secretion. The first identified inhibitor of 12-lipoxygenase, ML355, was evaluated in a mouse arteriole thrombus model. ML355 impaired thrombus formation and vessel occlusion with minimal effects on hemostasis [176]. Further studies are required to verify the efficacy and safety of this novel antiplatelet target.

## 6. Conclusions

Major advances in antiplatelet therapy have been accomplished over the last few decades. However, atherothrombotic events remain a leading cause of death worldwide. Incomplete protection and bleeding complications associated with the use of the currently available antiplatelet agents represent areas of development and deserve further investigation in order to appropriately manage CVD patients and provide better guidance in the search for new antiplatelet targets. Substantial research progress has been undoubtedly achieved, nevertheless, much remains to be done. 

## Figures and Tables

**Table 1:** Main pharmacological characteristics of the currently available antiplatelet agents.

Molecule	Drug Target	Route of Administration	Elimination Half-Life	Onset of Action after Loading Dose	Time to Platelet Function Recovery after Drug Discontinuation	Common Clinical Indication
Aspirin	Cyclooxygenase-1	oral *	15–20 min	~20 min ¶	5–7 days	ACS, CAD, PAD, stroke, TIA
Clopidogrel	P2Y_12_	oral	30 min #	2–6 h	7 days	ACS, CAD, stroke, TIA
Prasugrel	P2Y_12_	oral	30–60 min #	30 min	7–10 days	ACS
Ticagrelor	P2Y_12_	oral	7–9 h	30 min	3–5 days	ACS
Cangrelor	P2Y_12_	IV	3–6 min	≤5 min	30–60 min	ACS
Vorapaxar	PAR1	oral	5–13 days	-	4–8 weeks	PAD
Dipyridamole	PDE3/5	oral	10 h	-	-	Stroke, TIA
Cilostazol	PDE3A	oral	11–13 h	-	12–16 h	PAD
Iloprost	PGI_2_ analogue	IV	30 min	-	-	PAD
Eptifibatide	GPIIbIIIa	IV	2.5 h	≤15 min	4–8 h	ACS
Tirofiban	GPIIbIIIa	IV	2 h	20–40 min	4–8 h	ACS

* can be IV injected (in Europe) or administered as chewable tablets (in North America); ¶ few minutes (<5 min) if IV injected; # active metabolite; ACS, acute coronary syndrome; CAD, stable coronary artery disease; GP, glycoprotein; IV, intravenous; PAD, peripheral artery disease; PAR, protease-activated receptor; PDE, phosphodiesterase; TIA, transient ischemic attack.

**Table 2:** Novel antiplatelet agents under clinical development.

Name	Company	Type	Route of Administration	Target	Completed Clinical Trial	Reference
PZ-128	Tufts Medical Center	Pepducin	IV	PAR1	Phase I	[119]
BMS-986120	Bristol-Myers Squibb	Small molecule	oral	PAR4	Phase I	[120]
BMS-986141	Bristol-Myers Squibb	Small molecule	oral	PAR4	Phase I	_
					Phase II	_
Revacept	Advance Cor	Fusion protein	IV	GPVI ligand	Phase I	[121]
					Phase II	_
ACT017	Acticor Biotech	Antibody	IV	GPVI	Phase I	[122]
ARC1779	Archemix	DNA aptamer	IV	VWF	Phase I	[123]
					Phase II	[124]
AZD6482	AstraZeneca	Small molecule	IV	PI3Kβ	Phase I	[125]
					Phase I	[126]
Isoquercetin	Beth Israel	Small molecule	oral	PDI	Phase I	[127]
	NHLBI	Small molecule	oral	PDI	Phase II/III	[128]

GP: glycoprotein; IV: intravenous; PAR: protease-activated receptor; PDI: protein disulfide isomerase; PI3Kβ: phosphoinositide 3-kinase-β; VWF: von Willebrand factor.
